# The Multiple Roles of EG-VEGF/PROK1 in Normal and Pathological Placental Angiogenesis

**DOI:** 10.1155/2014/451906

**Published:** 2014-05-15

**Authors:** Nadia Alfaidy, Pascale Hoffmann, Houssine Boufettal, Naima Samouh, Touria Aboussaouira, Mohamed Benharouga, Jean-Jacques Feige, Sophie Brouillet

**Affiliations:** ^1^Commissariat à l'Energie Atomique (CEA), DSV-iRTSV, 17 rue des Martyrs, 38054 Grenoble Cedex 9, France; ^2^Université Grenoble-Alpes, 38041 Grenoble, France; ^3^Institut National de la Santé et de la Recherche Médicale U1036 (INSERM U1036), Biologie du Cancer et de l'Infection, Laboratoire BCI-iRTSV, CEA Grenoble, 17 rue des Martyrs, 38054 Grenoble Cedex 9, France; ^4^CHU de Grenoble, Hôpital Couple Enfant, Département de Génétique et Procréation, Centre d'Aide Médicale à la Procréation, CS 10217, 38043 Grenoble Cedex 9, France; ^5^Service de Gynécologie-Obstétrique “C”, Centre Hospitalier Universitaire Ibn Rochd, Faculté de Médecine et de Pharmacie, Université Hassan II, Ain Chok, 1 rue des Hôpitaux-ex Banaflous, 20360 Casablanca, Morocco; ^6^Plateau Commun de Recherche, Unité de Culture Cellulaire, Faculté de Médecine et de Pharmacie, 19 rue Tarek Bnou Ziad, 20360 Casablanca, Morocco; ^7^Centre National de la Recherche Scientifique, UMR 5249, 38054 Grenoble Cedex 9, France

## Abstract

Placentation is associated with several steps of vascular adaptations throughout pregnancy. These vascular changes occur both on the maternal and fetal sides, consisting of maternal uterine spiral arteries remodeling and placental vasculogenesis and angiogenesis, respectively. Placental angiogenesis is a pivotal process for efficient fetomaternal exchanges and placental development. This process is finely controlled throughout pregnancy, and it involves ubiquitous and pregnancy-specific angiogenic factors. In the last decade, endocrine gland derived vascular endothelial growth factor (EG-VEGF), also called prokineticin 1 (PROK1), has emerged as specific placental angiogenic factor that controls many aspects of normal and pathological placental angiogenesis such as recurrent pregnancy loss (RPL), gestational trophoblastic diseases (GTD), fetal growth restriction (FGR), and preeclampsia (PE). This review recapitulates EG-VEGF mediated-angiogenesis within the placenta and at the fetomaternal interface and proposes that its deregulation might contribute to the pathogenesis of several placental diseases including FGR and PE. More importantly this paper argues for EG-VEGF clinical relevance as a potential biomarker of the onset of pregnancy pathologies and discusses its potential usefulness for future therapeutic directions.

## 1. Introduction 

The placenta is one of the most densely vascularized organs in the organism [1]. At term, it has developed a capillary network that is *≅*550 km in length and 15 m^2^ in surface area [1, 2]. During the course of 9 months, placental vascular network expansion is a dynamic process characterized by intravilli vasculogenesis followed by branching and nonbranching angiogenesis [3]. Vasculogenesis starts during the third week after conception and involves* de novo* formation of new vessels. This process is characterized by the formation of the first blood vessels from differentiation of pluripotent mesenchymal cells into haemangiogenic stem cells [4]. The subsequent step, angiogenesis, starts during the fifth week after conception and refers to the development of new vessels from preexisting vessels [4, 5]. From day 32 to week 25 after conception, haemangioblastic cords formed by vasculogenesis develop into a richly branched villous capillary bed by two mechanisms: elongation of preexisting tubes and lateral ramification of these tubes (sprouting angiogenesis). Around week 25, this process switches from branching to nonbranching angiogenesis [4, 5]. Nonbranching angiogenesis occurs in mid and late gestation and it is mainly characterized by endothelial cell proliferation leading to an increase in the surface of the endothelial tissue. These processes ensure the increasing supply of gas and nutrient for the growing fetus [4, 5].

For many years, morphological and functional diversity among endothelia were thought to result from vascular bed-specific response to ubiquitous and tissue-restricted mediators. In this context, several ubiquitous growth factors (i.e., vascular endothelial growth factor (VEGF) and basic fibroblastic growth factor (bFGF)), as well as numerous pregnancy-specific angiogenic factors (i.e., placental growth factor (PlGF) and human chorionic gonadotropin hormone (hCG)), have been reported to regulate either the intravilli or the fetomaternal angiogenesis [4, 5].

The existence of tissue-specific angiogenic factors has been postulated for many years [6–9] but it only recently received confirmation when such a factor, named endocrine gland-derived vascular endothelial growth factor/prokineticin 1 (EG-VEGF/PROK1), was finally characterized [10].

## 2. EG-VEGF/PROK1 in the Placenta 

### 2.1. EG-VEGF, a New Angiogenic Factor Highly Expressed in the Reproductive Organs

In 2001, a novel family of angiogenic mitogens, named the prokineticins, has been characterized with restricted expression profiles and selective endothelial cell activity [10]. This family is composed of two members, EG-VEGF/PROK1 and PROK2, with multiple roles in physiological and pathological conditions. Human EG-VEGF and PROK2 proteins exhibit 44% amino-acid identity and share the same G protein-coupled receptors (PROKR), termed PROKR1 and PROKR2 [11]. PROKR bind the peptide hormones EG-VEGF and PROK2, with PROK2 showing a moderately higher affinity than EG-VEGF for both receptors [11]. Although* prokr1* and* prokr2 *genes are located on two different chromosomes (2q14 and 20p13, resp.), they encode proteins that share 85% amino acid identity and that exhibit the greatest differences in the N-terminal domains [11]. The exact functions of each receptor are not fully elucidated, but recent data reported specificities of actions in the heart and the placenta, where PROKR1 is preferentially involved in proliferation and angiogenic processes and PROKR2 is mainly implicated in endothelial permeability [12–14]. The prokineticins show different patterns of expression and preferential sites of actions. PROK2 is mainly associated with the nervous system, whereas EG-VEGF is predominantly associated with the reproductive tract and the endocrine organs, including the ovary and the placenta [10]. In the last decade, many studies have shed light on the angiogenic roles of EG-VEGF in the reproductive organs. In the gonads, EG-VEGF has been reported to impact physiological and pathological angiogenic processes [15–18]. In endothelial cells isolated from steroidogenic tissues, EG-VEGF has been shown to promote proliferation, differentiation, survival, chemotaxis, and fenestration of capillary endothelial cells [10, 19, 20]. Interestingly, the effect of EG-VEGF on endothelial cells seems to be tissue specific as it has no effect on endothelial cells derived from brain capillary, aorta, umbilical vein, or cornea [10, 21].

### 2.2. Placental Expression of EG-VEGF/PROKR Throughout Pregnancy

EG-VEGF is the major prokineticin in the female reproductive tract. In the placenta, EG-VEGF and its receptors are highly expressed [10, 22–26]. EG-VEGF is mainly localized to the syncytiotrophoblast layer (ST) with a mild expression in the cytotrophoblast layer (CT) [22]. EG-VEGF receptor PROKR1 is abundant in the CT, the placental microvascular endothelial cells (HPEC), and the Hofbauer cells (Ho), whereas PROKR2 is expressed by ST, HPEC, Ho, and extravillous trophoblasts (EVT) [22–25]. EG-VEGF and its receptors show a dynamic profile throughout pregnancy. In the placenta, EG-VEGF, PROKR1 and PROKR2 are predominantly expressed during the first trimester of pregnancy. During early pregnancy, EG-VEGF/PROKR1 peaks at 8–11 weeks of gestation (wg) and then gradually decreases by the end of the first trimester, whereas PROKR2 expression is maintained over the first trimester [22, 23, 26]. In nonpregnant women, circulating EG-VEGF levels are around 50 pg/mL [23]. During pregnancy, these levels significantly increase fivefold during the first trimester (*≅*250 pg/mL) and then gradually decrease to reach those observed in nonpregnant women by the end of the second trimester of pregnancy [23].

### 2.3. EG-VEGF/PROKR System and Placental Development

EG-VEGF is directly involved in the growth of the placental villi with multiple actions on various cell types. This factor is mainly produced by the ST [22, 23] and acts on the adjacent CT to increase their proliferation [27] at the expense of their differentiation to ST. This phenomenon contributes to the overall growth of the placental villi, an important aspect of placental development during the first trimester of human pregnancy. Moreover, EG-VEGF promotes the proliferation of anchoring trophoblasts and inhibits early EVT migration and invasion. In first trimester human placenta, anchoring trophoblastic plugs obstruct the spiral arteries and prevent maternal oxygenized blood from entering into the intervillous space. This physiological process creates a local hypoxic environment indispensable for normal placental and fetal development. At the end of the first trimester, anchoring trophoblast generates multilayered columns of EVT that invade the uterine blood vessels and remodel the maternal spiral arteries from minimal-flow/high-resistance vessels into larger diameter vessels with low resistance and high flow. The contribution of EG-VEGF in the formation and maintenance of the trophoblastic plugs throughout the first trimester protects the fetoplacental unit from early oxidative stress against which the first trimester placenta is not equipped [28, 29]. Altogether, these data demonstrate that EG-VEGF is a new placental growth factor that contributes to ensure the maintenance of pregnancy during the first trimester of pregnancy.

## 3. EG-VEGF Control of Placental Angiogenesis

Beside its effects on the trophoblastic component of the placental villi, growing evidences established the involvement of EG-VEGF and its receptors in placental angiogenesis. The following paragraphs will discuss how EG-VEGF controls the two main types of placental angiogenesis, the intravilli and the fetomaternal interface one.

### 3.1. EG-VEGF Effects on Fetomaternal Angiogenesis

**Figure 1:**
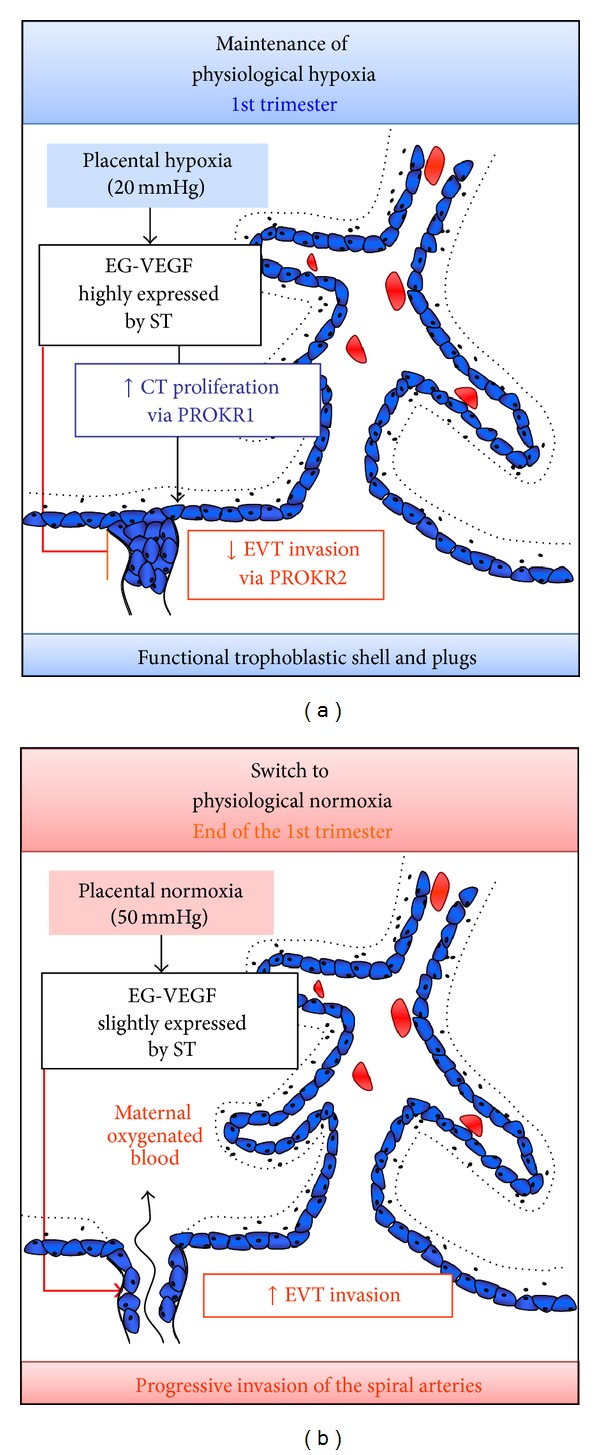
Proposed model of EG-VEGF-mediated effects on the fetomaternal angiogenesis during the first trimester of pregnancy. (a) and (b) represent cartoons of placental villi with EG-VEGF expression and actions on CT and EVT. (a) During the first trimester, EG-VEGF increases CT proliferation via PROKR1 activation and inhibits EVT invasion via PROKR2 activation. EG-VEGF/PROKR actions participate actively in trophoblastic shell and plugs constitution and contribute to the maintenance of physiological hypoxia during the first trimester of pregnancy. (b) At the end of the first trimester, EG-VEGF secretion declines. This contributes with other factors to EVT invasion and to the establishment of the fetomaternal circulation.

Trophoblastic invasion of spiral maternal arteries and decidua is the key process that establishes the fetomaternal circulation by the end of the first trimester of pregnancy. This process is known to be temporally and spatially controlled. Key studies from our group showed that EG-VEGF is a negative regulator of human EVT invasion. This statement was based on the demonstration that EG-VEGF inhibits EVT migration and invasion in HTR-8 cells (an extravillous trophoblastic cell line) and in first trimester villous explant culture systems and the demonstration that EG-VEGF inhibits HTR-8 cells organization into tube-like structures [23]. These data strongly suggest that EG-VEGF acts as an inhibitor of trophoblast differentiation towards an invasive phenotype and are consistent with a model of normal placentation in which downregulation of EG-VEGF expression at around 11 wg promotes differentiation of EVT. Therefore, the decrease in EG-VEGF circulating and placental levels at the end of the first trimester could contribute, with other factors, to extravillous trophoblast (EVT) invasion and to the establishment of fetomaternal circulation (Figure 1).

### 3.2. EG-VEGF Effects on Intravillous Angiogenesis

The placenta is composed of two types of endothelial cells: the microvascular endothelial cells (HPEC for human placental endothelial cells), cells that lie in the fetal capillaries of chorionic villi, and the umbilical vein macrovascular endothelial cells (HUVEC). It is well established that the endothelial cells that comprise the vascular beds of specific tissues are extremely diverse and display numerous tissue-specific characteristics in their phenotypes, growth properties, functions, and ultrastructure such as the intercellular junctions or the presence of fenestrae (for reviews see [30–32]). In accordance with these data, HPEC clearly differ from HUVEC in their phenotype and physiological functions [33–35]. HPEC show a spindle-shape that largely differs from the more polygonal phenotype of HUVEC [33–35]. HPEC grown* in vitro* secrete higher amounts of thromboxane and angiotensin II than HUVEC [34]. Furthermore, HPEC also show higher proliferative responses to tissue-restricted mediators (i.e., PlGF) in comparison to HUVEC [33–35]. Interestingly, ubiquitous angiogenic factors (i.e., FGF-2 and VEGF-A) exhibit similar effects on HPEC and HUVEC, suggesting that some tissue-restricted factors might contribute to endothelial singularity [6, 7, 33–35].

**Figure 2:**
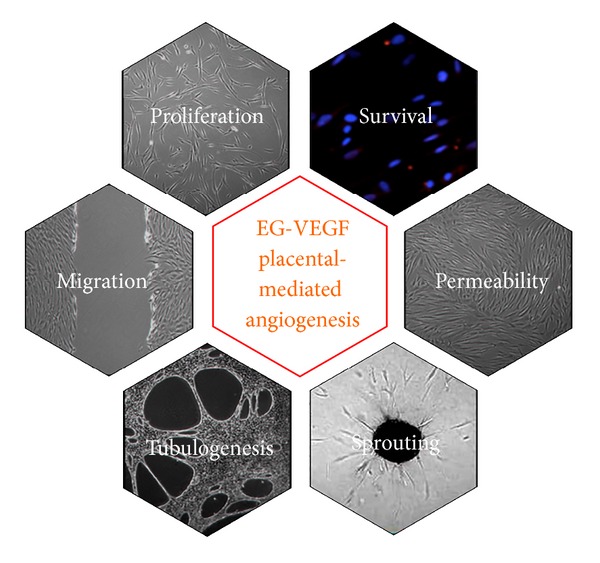
EG-VEGF is a new placental angiogenic factor. It controls placental growth via its multiple actions on endothelial cells within the chorionic villi.

In 2010, the angiogenic effects of EG-VEGF have been investigated in HPEC and HUVEC. Interestingly, EG-VEGF displayed specificity towards distinct vascular beds with major effects on HPEC-mediated angiogenesis (Figure 2). EG-VEGF increased HPEC proliferation, migration, tube-like formation, and sprouting, without affecting HUVEC-mediated angiogenesis. Both EG-VEGF receptors are expressed* in vivo* by placental HPEC and HUVEC. Quantification of PROKR1 and PROKR2 protein levels in endothelial cell primary cultures revealed larger expression of both receptors in HPEC than in HUVEC. This difference suggests a higher sensitivity of HPEC for EG-VEGF. Altogether, these data confirm the two distinct endothelial identities of HPEC and HUVEC and stress the importance to investigate placental angiogenesis with appropriate microvascular endothelial models.

The understanding of the mechanisms underlying placental angiogenesis was significantly improved by the use of* in vitro* models using appropriate endothelial cell cultures. In the last decades, numerous two- and three-dimensional assays helped to bring new insight into the understanding of EG-VEGF-mediated placental angiogenesis.

#### 3.2.1. Investigation of EG-VEGF Angiogenic Roles Using 2D-Primary Culture Models

*(i) Placental Endothelial Cells Primary Culture*. HPEC can be successfully isolated from the placental microvasculature by enzymatic perfusion of the placenta [21, 34] or from digestion of placental tissues [36–40]. Despite growing evidence demonstrating placental endothelial heterogeneity, HUVEC are still the most commonly used cell type for angiogenesis studies [8, 41]. Nevertheless, their above-mentioned differences in phenotype, gene expression, and physiology substantiate that microvascular endothelial cells are the unique model to use to investigate placental angiogenesis.

*(ii) EG-VEGF Effect on HPEC Proliferation and Survival.* Using complementary 2D-models, recent experiments have established the positive effect of EG-VEGF on HPEC proliferation and survival [21]. HPEC proliferation has been shown to be stimulated under EG-VEGF treatment, as assessed by [3H]-thymidine incorporation and Ki-67 staining [21]. EG-VEGF was also shown to promote endothelial survival as evidenced by decreased caspase-3 activity [21]. These results demonstrate that EG-VEGF is a new mitogenic and prosurvival factor for microvascular endothelial cells of the placenta [21]. 

*(iii) EG-VEGF Effect on Placental Endothelial Cell Migration and Tubulogenesis*. Using a quantifiable 2D-model of tubulogenesis, we established that EG-VEGF strongly promotes HPEC morphogenesis into tube-like structures [42–44]. Using a monolayer wound-healing assay, EG-VEGF effect on HPEC migration has been investigated. This assay is one of the earliest developed methods to study directional cell migration* in vitro *[45]. Our data demonstrates that EG-VEGF significantly increased the migration of HPEC in this model [21]. 

*(iv) EG-VEGF Effect on Placental Endothelial Permeability. *In the placenta, the microvascular endothelium is known to form a selective permeable interface that participates in the fetomaternal transports of solutes and nutrients. Therefore, the maintenance of a semipermeable endothelium is critically important for the development of the fetus. Placental microvasculature is not static and can be modulated by exposure to specific stimuli that affect intracellular permeability and paracellular transport. Using the HPEC model, we demonstrated that EG-VEGF increased both transendothelial permeability and paracellular transport [21]. Measurement of the ion flux through the primary HPEC monolayer was evaluated by transendothelial electrical resistance (TEER) measurement [21]. The use of siRNAs and blocking antibodies demonstrated that PROKR2 was specifically mediating EG-VEGF effects on cell permeability. In addition, the effect of EG-VEGF on the paracellular transport was also investigated by measuring [3H]-mannitol transport through HPEC monolayer. We observed that EG-VEGF almost doubled the [3H]-mannitol transport capacities of HPEC [21]. These results imply that EG-VEGF controls not only placental angiogenesis but also some physiological properties of placental vasculature such as permeability and transport of solute molecules. Altogether, the results suggest that EG-VEGF acts as a vascular bed-specific angiogenic factor providing an optimal vascular supply during human pregnancy.

#### 3.2.2. Investigation of EG-VEGF Angiogenic Roles Using 3D-Primary Culture Models

In addition to two-dimensional cell culture systems, 3D-models have also been employed to investigate EG-VEGF effect on placental angiogenesis. 

*(i) EG-VEGF Effect on Intravilli Vascularization Using Placental Explant Model. *Explants of human placenta are commonly used to study many tissue functions including cellular proliferation and differentiation [46]. In this system, EG-VEGF has been described to increase the number of differentiated endothelial cells (CD31+) within the villous tissue, suggesting an increase in the vascularisation within the placental villi [46]. This result is consistent with its proliferative and prosurvival effects observed in the 2D-primary microvascular cells model [21]. 

*(ii) EG-VEGF Effect on Endothelial Cell Sprouting. *The 3D endothelial spheroid model was used to study the role of EG-VEGF on placental endothelial cell sprouting. EG-VEGF significantly increased HPEC sprouting in a dose-dependent manner. Importantly, EG-VEGF treatment has a stronger effect than VEGF on HPEC sprouting [21]. The use of 3D-endothelial spheroid models confirmed the positive effect of EG-VEGF on placental angiogenesis previously reported using 2D-models. The use of siRNAs and blocking antibodies demonstrated that the effect of EG-VEGF on HPEC sprouting was specifically mediated by PROKR1. Such a selectivity of PROKR1 action in angiogenesis has also been found in other organs [12–14].

Using multifaceted strategies that included molecular, immunochemical, and functional approaches, several recent publications have shed lights on EG-VEGF key roles in placental angiogenesis via its specific effect on microvascular endothelial cells proliferation, migration, survival, tube organization, sprouting, permeability, and paracellular transport. Altogether, these findings imply that EG-VEGF might act in concert with other angiogenic factors to coordinate series of events that ensure the success of placental vascular development.

### 3.3. EG-VEGF, a Mediator of Placental Angiogenesis

During the last decade, many angiogenic actors have been described as regulators of the EG-VEGF/PROKR system, suggesting that EG-VEGF regulation of placental angiogenesis could be of direct or indirect form.

#### 3.3.1. Hypoxia: A Key Actor of Placental Angiogenesis and a Regulator of EG-VEGF

The human placenta develops in a low oxygen environment from the beginning of implantation to the end of the first trimester of pregnancy, due to proliferative trophoblast plugs within the maternal arteries that restrict blood flow into the intervillous space. This physiological hypoxia plays a key role in the modulation of the expression of several angiogenic factors [47, 48], including EG-VEGF. Numerous studies demonstrated that EG-VEGF is upregulated by hypoxia suggesting that this cytokine might mediate some of its angiogenic effects.

#### 3.3.2. Human Chorionic Gonadotropin (hCG): A Pivotal Hormone in Placental Angiogenesis That Increases EG-VEGF/PROKR System

Increasing evidence suggests that angiogenic effect of hCG on placental endothelial cells could be mediated by prior induction of EG-VEGF [49–51]. EG-VEGF and hCG are mainly secreted by the syncytiotrophoblast layer and exhibit similar patterns of expression with a peak around 8–10 wg. Recent findings demonstrate a new physiological regulation of EG-VEGF/PROKR system by hCG during the first trimester of pregnancy [51]. Using placental explants and primary trophoblast cultures, it has been established that hCG significantly increases EG-VEGF mRNA synthesis and protein secretion via the activation of the cAMP and protein kinase A signaling pathway [51]. HCG also induces mRNA and protein expression of PROKR1 and PROKR2 in first trimester human placenta [51]. These results reveal a new role for hCG in human placentation through its stimulation of the EG-VEGF/PROKR system and might explain the peak of expression of EG-VEGF and its receptors during the first trimester of pregnancy (8–11 wg). Moreover, EG-VEGF/PROKR regulation by hCG strongly suggests that some of the angiogenesis effects of hCG on placental villi might be mediated by EG-VEGF [49–51]. HCG is involved in many important functions in placental angiogenesis, including HPEC proliferation and sprouting [49, 50, 52].

#### 3.3.3. MAPK and PI3K/AKT: Key Signaling Pathways of EG-VEGF Angiogenesis

The MAPK and PI3K/AKT signaling pathways are highly involved in angiogenesis [53–57]. They play an essential role in the formation of normal blood vessels during development via their direct effects on endothelial cell proliferation, survival, differentiation, migration, and angiogenesis and contribute indirectly to the induction of angiogenesis by increasing the expression of numerous angiogenic factors such as VEGF, nitric oxide, and angiopoietin [53–57]. In HPEC, EG-VEGF induces a strong phosphorylation of MAPK and AKT proteins [21]. These data confirm the involvement of EG-VEGF in HPEC migration and survival and highly suggest its contribution in the induction of others angiogenic factors. Further investigations are required to determine the veracity of this hypothesis.

#### 3.3.4. IL-8: A Crucial Placental Angiogenic Factor Upregulated by EG-VEGF

Using third trimester placental explant model, a recent study has demonstrated IL-8 induction by EG-VEGF, potentially via activation of PROKR1 [24]. IL-8 is an important placental angiogenic factor that promotes endothelial cell chemotaxis and proliferation [58]. This cytokine is expressed in the human placenta throughout pregnancy and facilitates vascular permeability [59, 60]. Moreover, IL-8 is upregulated by HIF1*α* and is increased in conditions characterized by pathological angiogenesis such as placental vascular insufficiency [39, 61] and preeclampsia [62].

Altogether, these results demonstrate that EG-VEGF can directly and/or indirectly control placental angiogenesis. Hence, deregulations in EG-VEGF and/or its receptors could well be associated to vascular-associated pathologies during pregnancy.

## 4. Role of EG-VEGF in Pregnancy-Related Pathologies

It is well established that placental development depends on controlled growth, invasion, and differentiation of the trophoblast cells and on an adequate vascular development [63]. Hence, placental angiogenesis is highly linked to fetoplacental growth and fetomaternal exchanges. Abnormal angiogenesis has been associated with different pregnancy-related pathologies such as ectopic pregnancy, recurrent pregnancy loss (RPL), gestational trophoblastic diseases (GTD), preeclampsia (PE), and fetal growth restriction (FGR). EG-VEGF/PROKR expressions vary across normal pregnancy and in complicated pregnancies [21–23, 27, 51, 64, 65]. Recent studies have established correlations between abnormal EG-VEGF expression and pregnancy-specific diseases, ranging from miscarriage to intrauterine growth restriction and preeclampsia. These results strongly suggest that EG-VEGF deregulation could be associated with adverse pregnancy outcomes.

### 4.1. EG-VEGF in Recurrent Pregnancy Loss (RPL)

Recently, several publications demonstrated the involvement of EG-VEGF and its receptors in the etiology of RPL [66–68]. This pathology is widely attributed to chromosomal aneuploidy in the conceptus and/or to a deregulation in the expression of uterine factors. In the last decade, histological and ultrasound studies illustrated a link between recurrent miscarriage and abnormal vascularization in the placental bed, suggesting that early disturbance in placental vascular development might contribute to the pathogenesis of miscarriages [69–71]. In 2010, EG-VEGF receptor gene polymorphisms and haplotypes have been associated with RPL [67]. These data advocate that a deregulation in EG-VEGF-mediated signaling pathways could affect placental angiogenesis contributing to the pathogenesis of RPL. Further investigations are required to validate this hypothesis.

### 4.2. EG-VEGF in Gestational Trophoblastic Diseases (GTD)

Recent data have shown that maternal circulating levels of EG-VEGF are increased in patients undergoing molar pregnancies, a severe form of the gestational trophoblastic disease (GTD) [72]. GTD includes a wide spectrum of pathologies ranging from partial/complete hydatidiform moles to gestational trophoblastic tumors. Increasing data report a poor placental vascularization during the first trimester of GTD [23, 72–74]. EG-VEGF controls numerous angiogenic processes during the first trimester of pregnancy [64] and it is significantly increased by hCG [51], a hormone that is also highly upregulated in GTD. Altogether, these results suggest that EG-VEGF increased circulating levels reported in GTD could be a consequence of hCG deregulation and propose that its angiogenic effects might contribute to the pathogenesis of GTD during the first trimester of pregnancy. Further investigations are ongoing to identify the participation of EG-VEGF in the development of gestational trophoblastic diseases and to investigate the potential use of this placental specific factor for early diagnosis and treatment of GTD.

### 4.3. Preeclampsia (PE)

Recent publications report EG-VEGF and PROKR1 deregulations in PE and suggest their involvements in the development of this pregnancy-related pathology [23, 75]. PE is a systemic syndrome that is characterized by hypertension and proteinuria that appears around 22 weeks of gestation. PE affects approximately 5-6% of pregnancies worldwide accounting for nearly 18% of maternal deaths [76, 77]. The etiology of PE remains largely unknown but increasing evidences suggest that it originates from abnormal placentation. In the fetomaternal unit, PE is marked by insufficient trophoblast invasion and poor maternal spiral artery remodeling [78]. Further investigations pointed out the potential involvement of angiogenic factors and their receptors in PE development and stressed their potential importance in the prediction of its occurrence [79]. For instance, increased expression of soluble Flt1 and soluble endoglin in the maternal circulation weeks before the onset of PE has been reported and suggested as predisposing factors of the disease [79]. Recently, several findings strongly suggest that deregulation of EG-VEGF expression in the placenta might be associated with the development of PE [21–23, 27, 51, 64, 65]. In 2008, we reported a significant increase in EG-VEGF levels in the sera of third trimester PE patients as compared to age-matched controls [23]. More recently, a significant decrease in EG-VEGF mRNA expression has been reported in PE placentas [75], suggesting that local expression of EG-VEGF could be impaired at the transcriptional level in PE placentas. Determining the impact of these deregulations on placental development is difficult, as the relationship between maternal EG-VEGF circulating level and its local expression in the placenta remains unclear. EG-VEGF is also upregulated by hypoxia and hCG, two factors that are highly associated with the occurrence of PE [80–83]. The dynamic profile of EG-VEGF expression throughout pregnancy and its control of trophoblast invasion and placental angiogenesis strongly suggest that this cytokine contributes to the etiology of PE. Altogether, these results show that systemic and local EG-VEGF deregulation is associated with this pathology in the third trimester of pregnancy. However, we cannot conclude whether abnormal EG-VEGF levels are a cause or consequence of PE development. Further studies are needed to clarify whether EG-VEGF could be a predictive marker of PE.

### 4.4. Fetal Growth Restriction (FGR)

Optimal growth of the fetus throughout pregnancy depends on an adequate vascular network in the fetomaternal unit [84]. Therefore, abnormalities in placental microvascular development toughly compromise the supply of nutrients and hormones, leading ultimately to fetal growth restriction [85]. Interestingly, recent findings reported that EG-VEGF circulating levels were significantly higher in FGR patients during the third trimester of pregnancy [27]. These results were confirmed at the placental level where significant increases in EG-VEGF, PROKR1, and PROKR2 mRNA and protein expression were also found [27]. The authors proposed two hypotheses that could explain the association between EG-VEGF/PROKR system upregulation and the FGR condition. The first one proposed that EG-VEGF increased levels in FGR pregnancies could be a cause of the pathology, as sustained expression of EG-VEGF over the first trimester of pregnancy may compromise the spiral arteries remodeling and contribute to utero-placental hypoxia, a key parameter in the etiology of FGR [86, 87]. The second hypothesis proposed that FGR condition is caused by other predisposing factors that consequently increase EG-VEGF/PROKR system in the placenta and sera of the patients. This hypothesis is supported by recent* in vitro* experiments that demonstrate the strong upregulation of EG-VEGF and its receptors by hypoxia and hCG [22, 51], two parameters that are known to be increased in FGR [86–89]. Further studies are required to determine whether EG-VEGF/PROKR deregulation is a cause or consequence of FGR.

Altogether, these results clearly demonstrate that EG-VEGF and its receptors are closely associated to several pathologies marked by deregulated placental angiogenesis. These recent publications bring evidences for EG-VEGF association to key angiogenic processes and support the interest of new investigations on the predictive value of this factor in several pregnancy-associated pathologies including recurrent pregnancy loss, gestational trophoblastic disease, and placental pathologies associated with fetal growth restriction and/or preeclampsia.

## 5. Concluding Remarks 

Disruption in the balance of placental angiogenesis controlling factors may lead to abnormal vascular development and compromises the success of pregnancy. Alterations in numerous specific angiogenic-signaling pathways have been already described in pregnancy-related diseases. The multiple roles of EG-VEGF in the development of the chorionic villi argue for its clinical relevance as a diagnostic and/or prognostic marker for several placental diseases. The current challenge in the field of reproduction is to discover early biomarkers of abnormal placental angiogenesis to develop successful screening tests for pregnancy disorders. These biomarkers also represent potential new therapeutic targets to “rescue” placental vascular development and thus fetal growth in compromised pregnancies. In the last decade, compelling advances highlighted the pivotal role of EG-VEGF and its receptors in regard to their expressions, multiple roles, and regulations in normal and pathological human pregnancies. These fundamental and clinical results highly suggest that EG-VEGF might be a potential early marker for several pathologies including recurrent pregnancy loss, gestational trophoblastic diseases, FGR, and PE. Further studies are required to evaluate its potential relevance as an early marker of these pregnancy-associated pathologies, probably in combination with other predictive parameters such as uterine arteries blood flow measurements by Doppler ultrasound imaging [90].
